# The acute effect of wearable resistance placement on change of direction performance in elite netball players

**DOI:** 10.1002/ejsc.12081

**Published:** 2024-02-14

**Authors:** Chloe Ryan, Aaron Uthoff, Chloe McKenzie, John Cronin

**Affiliations:** ^1^ Sports Performance Research Institute New Zealand Auckland University of Technology Auckland New Zealand; ^2^ Athlete Training and Health Houston Texas USA

**Keywords:** change of direction, inertial measurement unit, netball, wearable resistance

## Abstract

The aim of this study was to determine the acute effects of wearable resistance forearm (WRf) loading versus shank (WRs) loading on change of direction (COD) performance in netball athletes. Ten elite female netball athletes (age: 24.9 ± 5.0 years, height: 180.1 ± 6.5 cm, weight: 81.3 ± 15.0 kg) participated in this within‐subject repeated measures study under three conditions: (1) no load (NL), (2) WRs and (3) WRf, both wearable resistance conditions loaded with 1% body mass on each limb. Athletes performed a modified 5‐0‐5 COD test with additional timing splits and inertial measurement units placed in their shoes. Total time was significantly longer for both WR conditions with a small effect compared to NL (*p* < 0.05, ES = 0.22–0.25). The greatest differences between WRs and WRf as compared to NL were in the acceleration phase with moderate effect sizes (0–2 m) (*p* < 0.05, ES = −0.67–0.79). Both loading conditions had moderate to large significant effects on peak deceleration (ES = 0.56–0.82) and maximum speed (ES = −0.50–0.60). No significant differences were observed between WR conditions. It appeared that WRs and WRs acutely affected COD performance and therefore might provide a potential training stimulus to elicit positive COD performance adaptations if used over an extended period of time. The choice of overload depended on the musculature that needed training.

## Highlights

- Both WRs and WRf significantly affected the total time and acceleration as compared to no load during a modified 5‐0‐5 COD test.
- Only WRs loading affected the deceleration split time compared to NL and WRf.
- No significant differences were observed during the COD phase.

## 1 INTRODUCTION

Many team sports, such as soccer, netball and rugby league, are characterized by high‐intensity movements, such as sprinting, rapid acceleration, deceleration, jumping and changing direction (Barber et al., 2016; Delaney et al., 2015; Taskin, 2008). Of these athletic movements, change of direction (COD) ability is considered one of the most essential for successful participation and performance in overground team sports (Dos’Santos et al., 2018; Nimphius et al., 2013). Netball is played on a 30.5‐m by 15.25‐m court, divided into equal thirds. Different playing positions are restricted to certain areas of the court, with some athletes (goal shoot and goal keep) only having a small space to move in. Netball is a sport characterized by sharp changes of direction to break free from an opponent in order to receive the ball (Steele, 1990). During elite netball matches, athletes can perform up to 63 ± 7.6 COD maneuvers during a 60 min match (Fox et al., 2014); thus, it would seem important for these athletes to have good COD ability and speed.

Previously, different training methods have been used in an attempt to enhance COD performance (Rydså & van den Tillaar, 2020). The most commonly used training methods are plyometric (ES = 0.60–3.50) (Asadi et al., 2016; Bogdanis et al., 2019; Jlid et al., 2019), traditional resistance (ES = 0.26–1.94) (Arazi et al., 2014; Hammami et al., 2017; Keller, Koob, Corak, von Schöning, & Born, 2020) and combination training (ES = 0.41–0.66) (Faigenbaum et al., 2007; Ramirez‐Campillo et al., 2018). These training methods have potentially shown a positive effect on COD performance (Falch, Rædergård, & van den Tillaar, 2019); however, a majority of the subjects were of young chronological age or low training age. Therefore, more experienced athletes may require greater specificity and individualization included in their strength and conditioning programme to elicit a positive effect in COD performance (Zatsiorsky et al., 2020). Some of the limitations of gym‐based trainings are that many of the exercises are performed bilaterally and are vertically orientated; however, movements such as sprinting and COD are primarily unilateral movements, which require vertical, horizontal, and lateral force production (Cleary Dolcetti et al., 2019). Implicit in the principle of specificity is that adaptations are specific to the nature of the training stress (Young, 2006). Because COD is a complex movement that requires a combination of accelerative, decelerative and direction change ability, a more movement specific approach may be of greater benefit compared to more traditional approaches such as plyometrics or resistance training.

One training method that allows for specific transference to occur is wearable resistance training (WRT). WRT involves an external load being applied to segments of the body during movement and is an example of the application of the concept of training specificity (Dolcetti et al., 2019). Wearable resistance involves the use of relatively light loads (1%–5% body mass) attached to wearable garments such as a vest to overload linear ground reaction forces, or distal segments of the body such as the shank and forearm, to overload rotational kinetics during sport‐specific movements such as sprinting or COD (Macadam, Cronin, & Feser, 2019; Macadam et al., 2021). Greater rotational kinetics may increase strength across the kinetic chain, and thereby improve sporting actions which require sequencing of multiple body segments, such as acceleration, deceleration and COD. To determine whether this training technique can be used longitudinally to improve COD performance, the acute effects of this loading strategy must be determined.

Rydså and colleagues (2020) examined the acute effect of different lower limb WR placements and various load on COD ability in male soccer players. The COD test consisted of 90° and 45° split times. The authors reported a large effect for different WR placements (ES = 1.4–2.0), with shank loading producing a longer total time and split times compared to thigh loading across all loading strategies. The largest total time significant (*p* < 0.05) difference was reported for 3% and 5% of body mass loading for shank and thigh, respectively (Rydså, 2020). Li et al. (2020), investigated the acute effects to COD performance during a 45° cut maneuver, in male soccer athletes, when an additional 5% body mass was attached to the torso. No significant differences between loaded and unloaded conditions in COD angle, approach speed, braking time, propulsive time, contact time and COD completion time were reported. A small increase in kinetics such as relative peak vertical propulsive ground reaction force (GRF) (*p* = 0.11, ES = 0.41) and relative peak braking GRF force (*p* = 0.22, ES = 0.38), were reported, which may be important kinetic stimuli for potential COD adaptation. To the authors knowledge there is no research that focuses on the acute effects of WR on COD performance in female athletes or netball athletes. Furthermore, researchers have focussed on the effects of lower body and trunk loading on COD performance; however, it has been reported that forearm loading provides a movement specific overload during sprinting (Macadam, Simperingham, & Cronin, 2019; Uthoff et al., 2021) and therefore may potentially provide a specific overload during COD tasks. Therefore, the aim of this research was to determine the acute effects of forearm loading versus shank loading on COD performance in elite female netball athletes.

## 2 METHODS

### 2.1 Experimental approach to the problem

A cross‐sectional within‐subject repeated measures design was used to determine the acute effects of WR on the kinematics and kinetics during a modified 5‐0‐5 COD test. Subjects performed the modified 5‐0‐5 COD test as fast as possible (3 trials on each leg), under three different conditions in a randomized order. The three conditions consisted of (1) shank loaded wearable resistance (WRs), with 1% body mass attached to each limb, (2) forearm loaded wearable resistance (WRf), with 1% body mass attached to each forearm and (3) no load (NL).

### 2.2 Subjects

Ten elite female netball athletes (age: 24.9 ± 5.0 years, height: 180.1 ± 6.5 cm, weight: 81.3 ± 15.0 kg) participated in this study. Athletes competed in the New Zealand netball premiership league and had a minimum of 6 years netball experience. Participants were required to be healthy and free of injury at the time of testing. All participants were provided with an information sheet and were required to fill out a written consent form prior to participating in this study. Participants were notified that they were free to withdraw from the study at any time.

### 2.3 Procedures

Prior to all testing sessions, subjects performed a 15‐min standardized warm up, consisting of lower body activation, such as banded walks and squats, vertical and horizontal (bilateral and unilateral) jumps, progressive sprints (5, 10 and 20 m) and COD drills, building the intensity up to maximum effort. Athletes were familiar with the modified 5‐0‐5 COD test, as they perform this on a regular basis as part of their in‐season training. For the modified 5‐0‐5 COD test, a modified set up was used, described by Ryan et al. (2021). Athletes began the test 0.5 m behind the first timing gate in a two‐point split stance, with their preferred foot forward. They could begin the test whenever they were ready. To ensure each athlete touched the line, the researchers observed each trial. Athletes were instructed to sprint 5 m and touch their foot on the COD line, perform a 180° on a specific leg and sprint back 5 m through the first timing gate. Three trials were performed on each leg for the 180° turn. Three minutes of passive rest was provided between trials to limit the effects of fatigue. If an athlete had a mistrial, they were given a retrial after 3 minutes of rest. The same procedure was repeated for all three testing sessions and conditions. One condition was randomly assigned to each subject each session. Sessions were exactly 7‐day apart and were held at the same indoor facility, at the same time of day. Participants wore the exact same clothing and shoes during each session to minimize changes in performance due to these factors.

### 2.4 Equipment

#### 2.4.1 Exogen wearable resistance

**FIGURE 1:**
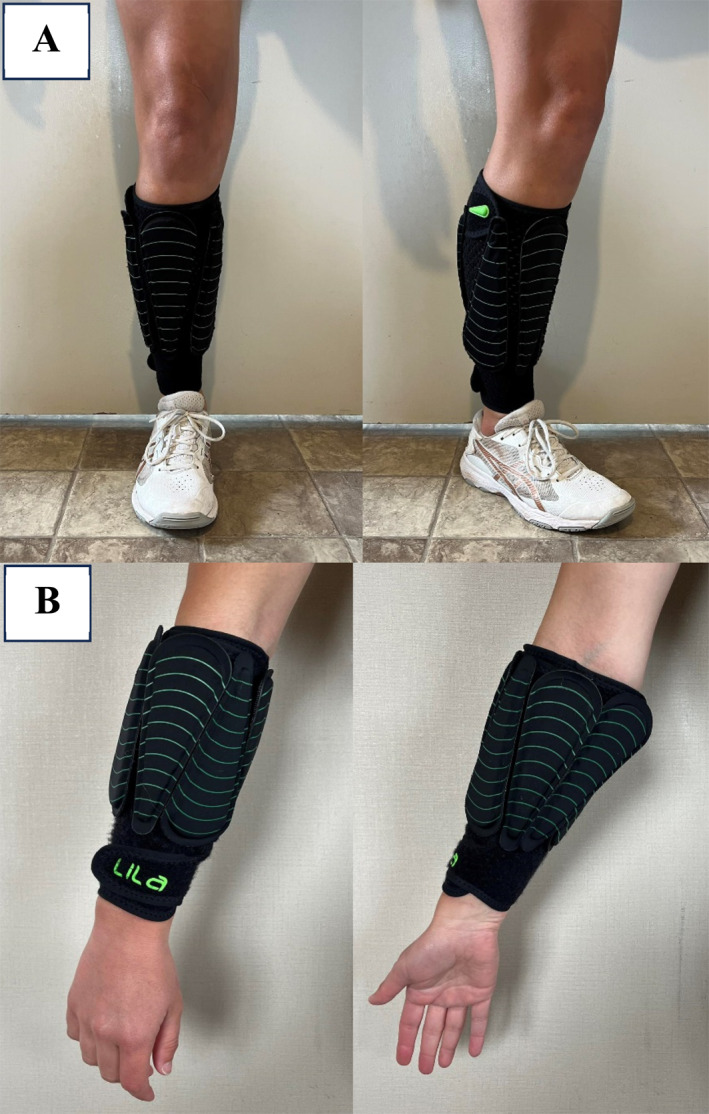
Illustration of 1% body mass loading with shank and forearm for 70 kg netball player.

For the WR shank loading, subjects were fitted with a pair of leg sleeves (Lila™, Sportboleh Sdh Bhd). Each subject was loaded with 1% of body mass to each limb using fusiform shaped loads of 50, 100, 200 and 300 g, which were attached to the garments via Velcro backing. Loading was calculated to the nearest 50 g. Loads were placed from most proximal to distal on the shank. The first load was attached vertically with the heaviest part placed anteriorly on the leg, while the next load was placed the opposite way (see Figure 1A). A similar loading configuration was used for the forearm loading condition (see Figure 1B). The loads were required to be placed vertically to ensure the full load could fit onto the athlete's shank and forearm.

#### 2.4.2 Inertial measurement unit

Plantiga IMUs (Plantiga Technologies, Vancouver, Canada; sampling frequency 416 Hz) were used to quantify the acute effects of different WR loading strategies on 5‐0‐5 COD performance. Plantiga insoles include 6‐axis IMUs (triaxial accelerometer and triaxial gyroscope) that are placed under each mid‐foot. Each IMU is small, durable (42 × 47 × 3.4 mm) and water and impact resistant. These insoles were fitted and placed in the participants' shoes prior to the warmup. Data was only collected during the modified 5‐0‐5 test via the Plantiga cloud accessed via a computer. Four different metrics were extracted from the IMU cloud and used for analysis. These metrics are calculated via the Plantiga software and have been reported to be reliable during a modified 5‐0‐5 COD test (ICC = 0.86–0.94, CV's = 2.3%–7.8%) (Ryan et al., 2023). Maximum speed was the highest speed achieved over the course of the modified 5‐0‐5 test and is calculated by averaging the speed of the left and right foot in the horizontal plane. Peak acceleration and deceleration metrics were also extracted from the cloud and is calculated from the rate of change of speed. Lastly, ground contact time (GCT) of the plant foot at the time of the turn was extracted for each trial. Plantiga accounts for drift with zero‐velocity detection, wherein when the foot is stationary, that is, on the ground, the IMU recalibrates.

#### 2.4.3 Timing lights

Dual beam timing gates (Swift Performance Equipment) were also used to quantify COD performance. Gates were set at 0, 2 and 4 m to isolate the phases of the 5‐0‐5 COD test (acceleration, deceleration, 180° turn and reacceleration), a method previously used by Ryan and colleagues (Ryan et al., 2021). Timing gate height was set at 1 m, in approximate line with center of mass. This set up produced five different splits, as well as a total 5‐0‐5 COD performance time. These times corresponded to the different phases of the modified 5‐0‐5 COD test as previously described by Ryan et al. (2021).

### 2.5 Statistical analysis

All statistical analyses were performed using IBM SPSS statistical software package (version 27.0; IBM Corporation). Data was presented as mean ± standard deviation (SD). Normal distribution of the data was checked using the Shapiro–Wilk test. Preliminary analysis was done on no load condition between left and right legs, using a paired samples *t*‐test. There were no significant differences between left and right leg data; therefore, data was pooled for further analysis. A one‐way repeated measures ANOVA with a Greenhouse–Geisser correction was used to determine the statistical differences in timing light and IMU variables between conditions. If significant differences were detected, post hoc comparisons with Bonferroni corrections were applied to determine where the differences occurred. The level of significance was set at *p* < 0.05. Mean difference, percentage change and Hedge's g effect sizes were calculated with 95% confidence intervals (CI). Effect sizes were interpreted using the following criteria: trivial effect = ≤ 0.2, small effect = 0.21–0.49, medium effect = 0.5–0.79 and large effect = ≥ 0.8 (Cohen, 1998). The smallest worthwhile individual change (SWC), moderate worthwhile change (MWC) and largest worthwhile change (LWC) was calculated on the pooled SD of the no load condition and converted to a percentage for each performance variable, where changes were deemed small (0.2 × SD), moderate (0.6 × SD) or large (1.2 × SD) (Hopkins, 2010).

## 3 RESULTS

Descriptive performance for timing light variables under the different loading conditions and their comparisons are presented in Table 1. Total time was found to be significantly longer with a moderate effect (0.04 s; 1.45%; ES = 0.22–0.25) for both the WR conditions as compared to the no load condition. The difference in total times were most likely due to significantly longer times (*p* < 0.05) with small to medium effects for acceleration (split 1) and deceleration (split 2) for shank loading (0.02–0.03 s; 3.85%–5.50%; ES = 0.47–0.79) and acceleration (split 1) for the forearm loading (0.02 s; 3.70%; ES = 0.67). No significant differences were observed between WR conditions, except for deceleration (split 2) which had a small yet significant effect (0.02 s; 3.78%; ES = 0.44). No significant differences were observed during COD phase (split 3) between loading conditions; however, a small effect was observed for forearm loading compared to no load (0.02 s; 3.17%; ES = −0.27). Furthermore, no significant differences were reported between conditions for reacceleration (splits 4–5).

**TABLE 1:** Acute differences between loading conditions for timing light variables and IMU variables.

Timing light variables	Loading conditions (mean ± SD)			Mean difference (95% CI)			Effect size (95% CI)		
	NL	WRs	WRf	NL–WRs	NL–WRf	WRs–WRf	NL–WRs	NL–WRf	WRs–WRf
Split 1 (s)	0.53 ± 0.03	0.56 ± 0.04	0.55 ± 0.38	−0.03* (−0.54 to −0.1)	−0.03*	0.01	−0.79	−0.67	0.13
					(−0.05 to −0.00)	(−0.2–0.03)	(−1.45 to −0.25)	(−1.26 to −0.16)	(−0.28–0.55)
Split 2 (s)	0.51 ± 0.04	0.53 ± 0.03	0.51 ± 0.03	−0.02^†^	−0.00	0.02*	−0.47	−0.06	0.44
				(−0.03 to −0.01)	(−0.01–0.01)	(0.00–0.30)	(−0.83 to −0.17)	(−0.27–0.14)	(−0.10–0.86)
Split 3 (s)	0.62 ± 0.07	0.62 ± 0.06	0.64 ± 0.09	0.00	−0.02	−0.02	0.00	−0.27	−0.29
				(−0.04–0.04)	(−0.07–0.03)	(−0.7—0.02)	(−0.35–0.35)	(−0.73–0.15)	(−0.72–0.11)
Split 4 (s)	0.64 ± 0.04	0.64 ± 0.05	0.63 ± 0.03	−0.00	0.01	0.01	−0.02	0.22	0.22
				(−0.02–0.1)	(−0.03–0.05)	(−0.04–0.06)	(−0.22–0.18)	(−0.43–0.90)	(−0.50–0.97)
Split 5 (s)	0.42 ± 0.03	0.43 ± 0.03	0.43 ± 0.03	−0.01	−0.01	0.00	−0.46	−0.33	0.13
				(−0.03–0.00)	(−0.03–0.01)	(−0.03–0.03)	(−0.93 to −0.06)	(−0.85–0.16)	(−0.58–0.85)
Total time (s)	2.74 ± 0.17	2.78 ± 0.18	2.78 ± 0.18	−0.05†	−0.04†	0.01	−0.25	−0.22	0.03
				(−0.81 to −0.13)	(−0.07 to −0.01)	(−0.05–0.06)	(−0.45 to −0.10)	(−0.40 to −0.06)	(−0.09–0.16)
IMU variables									
Max speed (m/s)	5.29 ± 0.53	4.94 ± 0.55	4.99 ± 0.55	0.35^†^	0.30^†^	−0.05	0.60	0.51	−0.08
				(0.19–0.51)	(0.13–0.48)	(−0.25–0.15)	(−0.29–1.0)	(0.24–0.86)	(−0.28–0.10)
Peak acceleration (m/s^2^)	4.13 ± 0.26	3.95 ± 0.30	4.00 ± 0.39	0.18^†^	0.14	−0.05	0.60	0.37	−0.12
				(0.13–0.23)	(−0.04–0.31)	(−0.20–0.11)	(0.33–0.96)	(−0.00–0.80)	(−0.43–0.17)
Peak deceleration (m/s^2^)	2.58 ± 0.27	2.39 ± 0.33	2.35 ± 0.22	0.19^†^	0.22^†^	0.04	0.56	0.82	0.12
				(0.05–0.32)	(0.08–0.37)	(−0.14–0.21)	(0.21–1.0)	(0.33–1.45)	(−0.28–0.54)
Ground contact time (s)	0.34 ± 0.08	0.30 ± 0.05	0.32 ± 0.05	0.03	0.02	−0.01	0.47	0.30	−0.23
				(−0.01–0.08)	(−0.02–0.06)	(−0.04–0.01)	(−0.03–1.02)	(−0.12–0.77)	(−0.61–0.12)

Abbreviations: CI, Confidence Intervals; NL, No load; SD, Standard deviation; WRf, Wearable resistance forearm; WRs, Wearable resistance shank.

* *p* < 0.05, † *p* < 0.01.

In terms of the IMU variables (see Table 1) it appears that the shank loading and forearm loading had fairly similar effects on the variables of interest. There were small to moderate significant (*p* < 0.01) differences between no load and WR conditions for maximum speed and acceleration (WRs = 4.46%–6.84%, ES = 0.60 and WRf = 3.20%–5.84% and ES = 0.37–0.51). It appears that shank loading had a larger effect (ES = 0.60) on acceleration, whereas forearm loading had a larger influence on peak deceleration (ES = 0.82) compared to NL. Once more no significant differences between WR conditions were observed.

**FIGURE 2:**
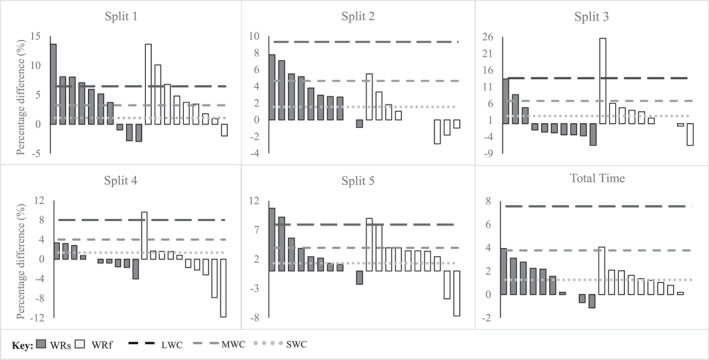
Individual time split responses to WR loading during the modified 5‐0‐5 COD test.

**FIGURE 3:**
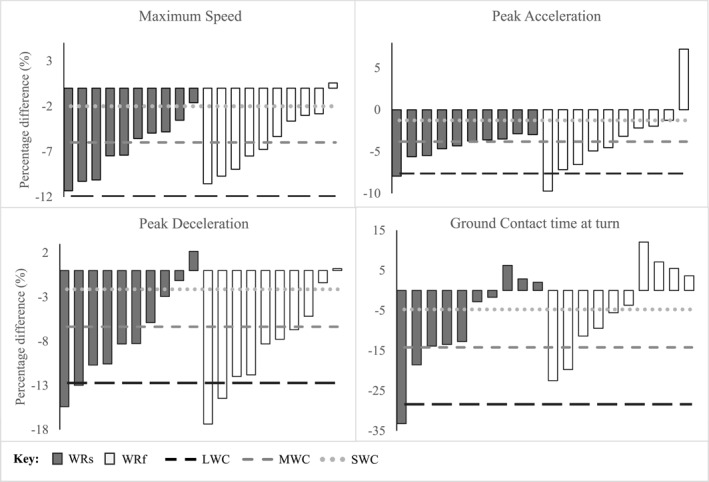
Individual responses to WR loading during the modified 5‐0‐5 COD test with IMU sensor.

Figures 2 and 3 provide graphical references illustrating the individual percentage changes relative to the SWC detected for each loaded condition compared to NL. As can be observed, 60% of the athletes were above the SWC for total time for both shank loading and forearm loading conditions. Similar patterns can be seen for acceleration (split 1) and reacceleration (split 5) where 70%–80% of the athletes were above the SWC. It was observed that 80% of the athletes were above the SWC for shank loading during deceleration (split 2), while only 30% were for forearm loading. In terms of IMU variables, 90%–100% of the athletes were above the SWC for maximum speed and peak acceleration, while 80% of the athletes were above the SWC for peak deceleration for both loading conditions.

## 4 DISCUSSION

The aim of this study was to determine the acute effects of forearm loading and shank loading compared to no load on 180‐degree COD performance in netball athletes. The main findings were (1) both WR loading conditions had a small to moderate significant effect on total time and the acceleration (split 1) time as compared to no load, (2) only shank loading had a small significant effect on the deceleration (split 2) time compared to no load and forearm loading, (3) no significant differences were found during the COD phase (split 3), (4) the different WR loading had fairly similar effects; however, peak acceleration was significantly affected by shank loading only as compared to no load, (5) no significant differences were observed between WR conditions on any of the IMU measures and, (6) in terms of individual responses, 60% of the athletes were above the SWC for total time, with similar results for acceleration (split 1) and reacceleration (split 5), while only shank loading appeared to affect the majority (80% above SWC) of the athletes during deceleration (split 2) compared to forearm loading. This seminal study provides practitioners with knowledge pertaining to the acute effects of shank and arm loaded WR on COD performance. Given the importance of a training stimulus to have a slight acute effect in performance in order to elicit chronic adaptation if the stimulus is used consistently with the correct dosage and adequate recovery, the use of WR may provide a potential training method for inducing adaptation during specific phases of the 5‐0‐5 COD test (Lambert, 2016).

Both forms of loading significantly increased total time during the modified 5‐0‐5 COD test compared to the no load condition. The authors expected there to be a small effect for both loading conditions, specifically during the linear phases (splits 1 and 4–5), as this would be where the athletes are moving the fastest and therefore creating the greatest rotational overload; however, trivial and small differences over the five splits led to small yet significant increase in total time (ES = 0.22–0.25). During the initial acceleration phase of the modified 5‐0‐5 COD test, both shank and arm loading of 1% body mass had a significant effect on performance. To the authors knowledge, this is the first study to examine the acute effects of shank and forearm WR on the initial acceleration of a COD test; however, previous researchers have explored the acute effects of different WR loading strategies on linear sprint acceleration (Feser et al., 2018; Macadam, Cronin, et al., 2019; Macadam et al., 2020; Macadam et al., 2021; Simperingham et al., 2022; Uthoff et al., 2020; Uthoff et al., 2021). Simperingham et al. (2022) found no significant acute effects on initial acceleration over 5 m using loads ranging from 3% to 5% body mass distributed across both the thigh and shank. The difference between these and the results of this study could be attributed to a combination of factors. Firstly, the physical determinants of rugby differ from netball athletes (Simpson et al., 2019; Thomas et al., 2019; Zabaloy et al., 2021). Secondly, the technologies used to quantify performance differed between studies, with Simperingham et al. (2022), using radar technology and a non‐motorized treadmill, whereas the current study used timing gates and IMU technology. Lastly, given the equation of rotational inertia (rotational inertia = mass x radius^2^), the relatively greater distribution of mass in the current study, that is, all load placed below the knee, may influence performance due to the greater rotational inertia and thus a greater overload (Dolcetti et al., 2019). With regards to arm loading, no significant acute effect during acceleration (0–10 m) were observed in the study of Uthoff et al. (2020), using forearm loads of 1% BM. Conversely Macadam et al. (2019) reported a small significant 2.1% difference in acceleration between forearm loaded (% BM) and no load (ES = 0.46), which were similar to the results reported in this study (3.5%, ES = 0.67).

With regards to the deceleration performance, shank loading was found to significantly increase time during this phase (i.e. split 2) compared to both the no load and forearm loaded conditions. To the authors' knowledge, no previous research has looked at a specific deceleration phase or metric during linear sprinting or COD whilst wearing shank or forearm loaded WR. It intuitively makes sense that shank loading had a larger overall effect on deceleration, as the load is placed distally from the axis of rotation (hip and knee joint) and directly overloads the specific joints and musculature such as the quadriceps and gastrocnemius used during deceleration (Andrews et al., 1977; Hewit et al., 2011). Though the arm has a far greater load placed on it in proportion to segment mass, the distance from the axis of rotation is smaller than that of the shank; therefore, it is possible that the shank loading has equal or greater rotational inertia compared to the forearm loading. The authors acknowledge that this is difficult to quantify, and all athletes will be different depending on anthropometrics. It is important to note that the population used in this research were above average height, given that they are elite netball athletes; therefore, the distance from the axis of rotation may be greater than that of an average population. Additionally, from a practical standpoint, it proved difficult to attach load greater than 1% body mass to the athletes who participated in this research, due to a smaller surface area compared to the shank.

There were no significant differences reported for the COD phase (i.e. split 3), which was expected as during this COD phase, there is minimal rotational kinetic energy as rotational velocity at the knee and hip is minimal; therefore, limb loaded WR would have its least effects. There was a small effect (ES = −0.27), however, when comparing arm loading to no load. The authors hypothesized that forearm loading may have had a greater acute effect on the COD split, due to the importance of the arm movement during COD movements. When an athlete plants their foot to perform a COD, their inside arm pulls in a backward direction as the outside arm moves forward to assist rotation of the body (Brown, 2003). Whereas during this COD phase, there is limited rotational movement occurring at the hip; therefore, it would be hypothesized that shank loading would have minimal effect on this phase.

No significant differences were reported for the reacceleration phases (i.e. split 4 and 5); however, there were significant differences reported for maximum speed measured with the IMU for both loaded conditions compared to the non‐loaded condition, but no differences were observed between loaded conditions. Maximum speed is likely to be occurring during the reacceleration phases, as this is when athletes have the largest distance to cover without the need to prepare for a COD, allowing them to reach their highest velocity. Additionally, only shank loading significantly affected peak acceleration as measured with the IMU compared to both WR loading conditions affecting the acceleration timing split. This is most likely explained by the greater rotational inertia (I) of the shank loading given that the WR mass was located a greater distance (r) from the hip axis of rotation as compared to WR forearm placement, that is., I = mr^2^ (Feser et al., 2018). Other potential reasons for the different acute effects could be attributed to technological differences, such as (1) whilst IMU is measuring at 416 Hz (416 samples per second) the timing lights only give information when the beams are broken, for example, ∼0.5 s, (2) measurements occurring at different body parts, that is, timing lights are broken when the center of mass passes through and breaks the beam; however, the IMU is measuring what is happening directly at the foot, and (3) the IMU peak acceleration is taken from one point (peak value), whereas the timing splits consider acceleration over a specified distance. Logically, it would appear that these two technologies are providing unique and independent information.

Though no significant differences were found between WR conditions on any of the measures, for timing light or IMU, except for the deceleration split (split 2), individual responses provide greater insight into the effects of each loading strategy. As shown in Figures 2, 60% of the athletes were above the SWC for total time, with similar results for acceleration (split 1) and reacceleration (split 5) for both WR loading conditions. However, shank loading appeared to affect the majority (80% above SWC) of the athletes during the deceleration split (split 2), increasing split times as compared to forearm loading (30% above SWC). Based on these graphical representations (Figures 2 and 3), it would appear that the shank loading significantly affected more athletes, that is, increasing split times and decreasing IMU variables (peak acceleration, peak deceleration and maximum speed) as compared to forearm loading.

In terms of previous studies, the authors have found limited research pertaining to the acute effects of WR during a COD task. One group of researchers investigated the acute effects of wearing 5% BM weighted vest; however, this was during a 45° cutting movement in male soccer athletes, with no significant differences reported (Li et al., 2020). Another research group investigated the acute effects of 1% body mass per leg attached to the shank and thigh during a 45° (cut) and 90° (turn) COD performance in male soccer athletes (Rydså & van den Tillaar, 2020). They reported significant total time differences (*p* < 0.05) in both shank and thigh loaded conditions compared to unloaded, with the shank loaded protocol resulting in slower, yet not significant, total times compared to thigh loading. It is difficult to make comparisons across studies due to numerous reasons. Firstly, the degree of COD were different, with Rydså and van den Tillaar (2020) reporting greater differences between interventions with the 90° turn compared to the 45° cut, which leads to the speculation that the larger the COD angle, the higher the overload of the WR. Secondly, the subjects used in the previous studies have both used male soccer athletes, whereas this study has used female netball athletes. How different loading strategies effect male and female athletes is not yet documented; therefore, it is difficult to make comparisons across sexes. Additionally, the physical determinants likely differ between field sports with large playing areas, versus court sports with relatively confined playing areas; therefore, further research is necessary to elucidate acute adaptations to WR for different sporting codes.

### 4.1 Limitations

A limitation of this study was the sample size and population used, namely elite level netball athletes, meaning only a small number of participants were able to be recruited. However, we would assume that if the WR loading strategies caused an acute effect for elite netball athletes, a similar or greater effect would be observed for developing players who are not as proficient at performing a 180° COD. Another limitation was that only one load (1% BM) was used in this study and greater loading, such as 1.5%–2% body mass on each limb, may induce larger effects on COD performance. However, further research is needed to confirm this, as greater load would result in slower movements, and therefore less kinetic energy, potentially resulting in negligible effects. Additionally, the researchers were unable to fit more than 1% body mass loading to each arm on the female netball athletes; however, further loading could be placed on the shank. Lastly, the authors did not capture videography alongside the other technologies used; therefore, it is difficult to determine how different loading parameters affected technique during the 5‐0‐5 test.

## 5 CONCLUSION AND PRACTICAL APPLICATIONS

Using the modified 5‐0‐5 COD test with the phase splits and IMU measurements allows coaches and practitioners to be more granular with their programing. For example, if an athlete has a poor acceleration split, targeted acceleration programing should be used. In terms of WR, it appears that both shank and forearm loading can be used to induce acute changes to phases of COD performance. However, practitioners can use different loading strategies to train different phases of the modified 5‐0‐5 test. For example, if an athlete has poor decelerative ability, shank loading could be used due to shank loading having the greatest significant effect on mean deceleration measured via the timing split and peak deceleration measured via the IMU. However, if an athlete is struggling with arm drive, or their COD phase, then forearm loading may be best. While both forms of load were observed to overload the initial acceleration phase, max speed and peak deceleration, shank loading also overloaded peak acceleration and deceleration performance prior to the COD phase. Therefore, if practitioners are limited to only being able to employ one loading protocol, shank loading would be recommended. This information provides practitioners with information regarding a new potential training stimulus that may elicit positive adaptations in COD performance if used over an extended period of time; however, training interventions are needed to confirm this hypothesis. Additionally, further research is needed to determine the acute effects of WR loading on COD performance across different COD angles, with the use of video to determine the effects on technique. Additionally further research is needed to determine the different responses between male and female athletes.

## CONFLICT OF INTEREST STATEMENT

No competing interest were disclosed.

## Data Availability

The data that support the findings of this study are available on request from the corresponding author. The data are not publicly available due to privacy or ethical restrictions.
